# Medical and Surgical Management of Endometriosis: A Narrative Review of Clinical Outcomes

**DOI:** 10.7759/cureus.107137

**Published:** 2026-04-16

**Authors:** Ashmitaa Srianand, Devi Priya R, Saumya, Pragati Sakhuja, Nikeeta Ashokrao Khanorkar, Sumit Sukla Das

**Affiliations:** 1 Department of Obstetrics and Gynaecology, Kauvery Hospital, Chennai, IND; 2 Department of Obstetrics and Gynaecology, ACS Medical College and Hospital, Dr. M.G. Ramachandran Educational and Research Institute, Chennai, IND; 3 Department of Obstetrics and Gynaecology, Government Medical College, Satna, Satna, IND; 4 School of Civil and Chemical Engineering, Manipal Institute of Technology, Manipal, IND; 5 Department of Obstetrics and Gynaecology, Government Medical College, Nagpur, Nagpur, IND; 6 Department of Obstetrics and Gynaecology, Agartala Government Medical College (AGMC), Agartala, IND

**Keywords:** endometriosis, fertility outcomes, medical management, quality of life, surgical management

## Abstract

Endometriosis is a chronic, estrogen-dependent, and heterogeneous gynecologic disorder associated with persistent pain, infertility, and substantial impairment in quality of life (QoL), necessitating individualized and evidence-based management. This narrative review synthesizes contemporary evidence on the clinical outcomes of medical and surgical management of endometriosis, with particular emphasis on pain control, fertility outcomes, disease recurrence, QoL, and treatment safety. A structured literature search of PubMed/MEDLINE, Embase, Scopus, and the Cochrane Library was conducted for studies published between 2015 and 2025, including randomized controlled trials, observational studies, and high-quality reviews reporting clinically relevant outcomes following medical and/or surgical interventions. Medical therapies, including hormonal suppression strategies, are consistently effective in reducing dysmenorrhea and chronic pelvic pain; however, they are limited by treatment-related adverse effects, symptom recurrence after discontinuation, and lack of sustained fertility benefit. Surgical management, particularly laparoscopic excision, demonstrates significant improvements in pain and spontaneous conception rates in selected patients; however, risks of recurrence, surgical complications, and reduction in ovarian reserve remain important considerations. Postoperative hormonal suppression is associated with prolonged recurrence-free intervals and improved long-term symptom control. Patient-reported outcome measures highlight meaningful improvements in QoL across treatment modalities but also underscore variability in long-term outcomes. Optimal management of endometriosis requires a multidisciplinary, patient-centered approach that integrates medical and surgical strategies, fertility preservation considerations, and long-term outcome monitoring to address the complex and recurrent nature of the disease.

## Introduction and background

Endometriosis is a chronic, estrogen-dependent inflammatory disease characterized by the presence of endometrial-like tissue outside the uterine cavity, most commonly affecting the pelvic peritoneum, ovaries, and deep pelvic structures [1]. It is among the most prevalent gynecologic diseases worldwide; however, its true epidemiology remains poorly understood due to diagnostic delays, reliance on invasive diagnostic procedures, and heterogeneity of clinical manifestations [2]. Although the prevalence of endometriosis is typically estimated at approximately 10% among women of reproductive age, it increases substantially among patients with chronic pelvic pain or infertility, indicating that a significant number of cases remain undiagnosed or unreported [3].

The pathophysiology of endometriosis is multifactorial and complex, involving hormonal imbalance, chronic inflammation, immune dysregulation, neuroangiogenesis (formation of new nerve fibers and blood vessels within lesions), and altered pain processing [2]. Classical theories such as retrograde menstruation (backflow of menstrual blood into the pelvic cavity) do not fully explain disease occurrence, particularly in distant or deep infiltrating lesions, suggesting the involvement of additional mechanisms such as coelomic metaplasia (transformation of peritoneal cells into endometrial-like tissue), stem cell differentiation, and genetic susceptibility [4]. More importantly, endometriosis exhibits significant biological and clinical heterogeneity, with variations in lesion histomorphology, anatomical distribution, molecular patterns, and symptom severity. This heterogeneity, similar to other chronic inflammatory and immune-mediated conditions, contributes to variability in disease occurrence and inconsistency in treatment outcomes [5].

Endometriosis is associated with a wide range of symptoms, most notably dysmenorrhea, chronic pelvic pain, dyspareunia, and infertility. Pain often persists despite treatment and is not always proportional to disease severity, highlighting the role of central sensitization (increased pain sensitivity due to central nervous system changes) and neuroinflammatory processes [4]. Infertility associated with endometriosis remains a significant clinical concern and is caused by disruption of normal pelvic anatomy, an inflammatory peritoneal environment, impaired folliculogenesis, reduced oocyte quality, and altered endometrial receptivity [6]. Endometriosis is not defined solely by physical symptoms but also by its impact on health-related quality of life (QoL) [7]. Women with endometriosis report higher rates of depression, anxiety, sexual dysfunction, and impaired social and occupational functioning [8]. These findings indicate a substantial psychosocial burden and support the need for multifaceted, patient-centered management approaches [9].

Endometriosis is managed using both medical and surgical approaches, which are often applied sequentially or in combination [3]. The primary objective of medical treatment is to suppress ovarian estrogen production and reduce inflammatory activity, thereby alleviating pain and slowing disease progression [2]. Common medical therapies include combined oral contraceptives, progestins, gonadotropin-releasing hormone (GnRH) agonists and antagonists, and levonorgestrel-releasing intrauterine systems. The most common surgical approach is laparoscopy, performed to excise or ablate endometriotic lesions, restore normal pelvic anatomy, and improve pain and fertility outcomes [7]. This approach is particularly indicated in cases of deep infiltrating endometriosis, endometriomas, or symptoms that are refractory to medical therapy; however, it is associated with risks of recurrence, surgical complications, and potential reduction in ovarian reserve [10].

Although several therapeutic options are available, there is no consensus on the optimal treatment strategy [6]. International guidelines differ in recommendations regarding treatment sequencing, duration of medical therapy, and indications for surgery, reflecting inconsistencies in the underlying evidence base [8]. In addition, diagnostic limitations, particularly in non-ovarian and deep disease, complicate patient stratification and outcome assessment [1]. Imaging modalities such as transvaginal ultrasound and magnetic resonance imaging (MRI) have improved diagnostic accuracy; however, variability in sensitivity and operator expertise continues to influence clinical decision-making [11].

**Figure 1:**
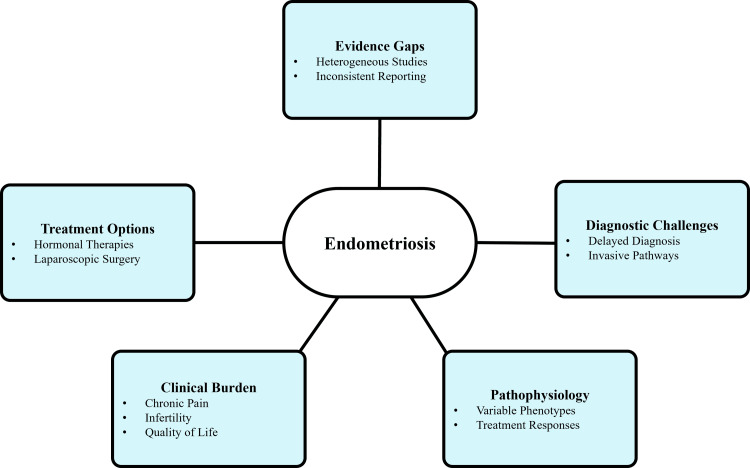
Disease complexity and evidence gap in endometriosis. Created by authors using Microsoft PowerPoint.

Significant heterogeneity in study design, outcome measures, and follow-up duration remains a major limitation in current research, restricting direct comparison of medical and surgical management strategies. Long-term outcomes, including recurrence patterns, fertility outcomes, and QoL measures, are inconsistently reported, with a predominance of studies focusing on short-term pain relief. Moreover, intervention-related adverse outcomes, such as hypoestrogenic effects of hormonal therapy, surgical complications, and impacts on ovarian reserve, are variably reported, limiting comprehensive risk-benefit assessment. The lack of standardized outcome reporting and limited integration of patient-centered measures restrict the translation of evidence into individualized clinical practice. These gaps highlight the need for harmonized research methodologies, standardized outcome measures, and multidisciplinary approaches to generate clinically meaningful evidence. Figure 1 illustrates disease heterogeneity, diagnostic challenges, and existing gaps in the assessment of therapeutic outcomes in endometriosis research.

Objectives of the review

The objective of this narrative review is to synthesize and critically evaluate contemporary evidence on the clinical outcomes of medical and surgical management of endometriosis. This review focuses on pain relief, fertility outcomes, recurrence rates, QoL, and treatment-related adverse events. By integrating findings across diverse treatment modalities, it aims to inform individualized, evidence-based clinical decision-making and identify gaps for future research.

## Review

Methodology

A comprehensive literature search was conducted in PubMed/MEDLINE, Embase, Scopus, and the Cochrane Library to identify studies evaluating medical and surgical management of endometriosis. The search covered publications from January 2015 to December 2025 and was limited to human studies published in English. Controlled vocabulary terms (MeSH/Emtree) and free-text keywords related to endometriosis, medical and hormonal therapies, surgical interventions, and clinical outcomes (e.g., “endometriosis,” “hormonal therapy,” “laparoscopy,” “fertility outcomes,” and “pain management”) were combined using Boolean operators. Reference lists of relevant articles were also manually screened to identify additional eligible studies. Efforts were made to include recent high-impact studies, including randomized controlled trials, meta-analyses, and large cohort studies addressing emerging areas such as GnRH antagonists, advanced imaging techniques, fertility preservation strategies, and long-term outcomes. Although this is a narrative review, the methodology followed a structured approach; however, no formal Preferred Reporting Items for Systematic reviews and Meta-Analyses framework or standardized risk-of-bias assessment tool (e.g., Cochrane Risk of Bias or Risk of Bias in Non-randomized Studies-Interventions) was applied.

A total of 45 studies meeting the inclusion criteria were included in the final synthesis, comprising randomized controlled trials, cohort studies, and high-quality observational studies. Study selection was performed using predefined inclusion and exclusion criteria, focusing on clinically relevant outcomes such as pain relief, fertility outcomes, recurrence, QoL, and treatment safety. Study selection was conducted in a stepwise manner, including initial screening of titles and abstracts followed by full-text assessment to determine eligibility based on predefined criteria. Due to heterogeneity in study designs, populations, and outcome measures, no quantitative synthesis or meta-analysis was performed. This approach was considered appropriate given the substantial heterogeneity in study design, populations, and outcome measures, which limited the feasibility of formal statistical pooling. Data were synthesized narratively using an outcome-based framework to ensure consistent interpretation across heterogeneous studies. Therefore, no additional statistical analysis or specialist statistical review was required.

Methodological quality and potential risk of bias were assessed qualitatively by evaluating study design, sample size, consistency of reported outcomes, and duration of follow-up, rather than applying a formal validated risk-of-bias tool. Risk of bias was assessed qualitatively as part of the evidence synthesis by evaluating study design, methodological rigor, and consistency of reported outcomes. This approach allowed inclusion of heterogeneous yet clinically relevant evidence while maintaining a critical and balanced interpretation of findings.

Outcome-based comparative assessment

Clinical evidence on endometriosis management is most effectively synthesized using an outcome-based framework that prioritizes treatment impact over intervention type [12]. The therapeutic effectiveness has been reported across the studies included in the present review across several clinically meaningful outcome domains [13]. The following comparative assessment is based on these domains, which include pain control, fertility outcomes, disease recurrence, QoL, and treatment safety.

The medical and surgical procedures are considered simultaneously within these outcome domains to enable balanced interpretation of their advantages and limitations [14]. This framework reflects modern clinical practice, where treatment approaches are often applied sequentially or concomitantly. Outcomes are influenced by disease severity, lesion characteristics, reproductive intent, and duration of follow-up. As the heterogeneity of study design, outcome definitions, and measurement tools is high, the synthesis does not focus on individual effect estimates but rather on the consistency of observed trends [15].

Within this framework, key outcome domains are evaluated systematically. Pain outcomes are assessed through dysmenorrhea, chronic pelvic pain, and dyspareunia to reflect the chronic and intervention-sensitive nature of symptoms [16]. Fertility outcomes are discussed in terms of spontaneous conception and assisted reproductive technology (ART), highlighting different clinical pathways and prognostic implications [17]. Recurrence is evaluated based on symptom relapse and disease re-emergence, particularly in relation to postoperative or post-treatment hormonal suppression [18]. Patient-reported outcome measures (PROMs) are used to synthesize QoL outcomes across functional, emotional, and sexual domains that are not adequately captured by clinical endpoints. Safety outcomes include adverse effects, treatment tolerability, surgical morbidity, and changes in ovarian reserve [19].

This structured, outcome-based narrative synthesis enables integrated interpretation of the available evidence while improving clarity and consistency across sections. It also provides a coherent foundation for the detailed evaluation of medical and surgical management strategies that follow (Table 1).

**Table 1:** Comparative evaluation of endometriosis management. QoL: quality of life

Outcome domain	Scope of evaluation	Considerations	References
Pain control	Dysmenorrhea, chronic pelvic pain, dyspareunia	Symptom chronicity, differential response patterns, persistence after treatment	[16]
Fertility outcomes	Spontaneous conception and assisted reproductive technology	Reproductive intent, disease severity, postoperative pathways	[17]
Disease recurrence	Symptom relapse and disease reappearance	Follow-up duration, completeness of intervention, role of hormonal suppression	[18]
QoL	Patient-reported physical, emotional, and sexual well-being	Functional impact beyond clinical symptom scores	[15]
Treatment safety	Medical and surgical adverse effects	Tolerability, morbidity, and effects on ovarian reserve	[19]

Pain outcomes following medical therapy

Dysmenorrhea is one of the most common and debilitating symptoms of endometriosis, and medical therapy plays a central role in its management [20]. Hormonal therapy reduces menstrual flow and suppresses ovulation, resulting in significant improvement in cyclical pain [13]. Although dysmenorrhea shares mechanistic features with primary dysmenorrhea, endometriosis-associated pain is typically more severe and persistent, requiring sustained hormonal suppression rather than short-term symptomatic management [20]. Therapeutic exercise, as part of adjunctive non-pharmacologic therapy, may contribute to pain reduction through modulation of inflammatory and neuromuscular pathways; however, its effectiveness in endometriosis-specific pain syndromes remains limited [21].

In addition to dysmenorrhea, chronic pelvic pain represents a major clinical challenge in endometriosis. Chronic pelvic pain is a multifactorial manifestation that often persists beyond menstruation [19]. Medical therapies targeting hormonal and inflammatory pathways provide moderate pain reduction; however, responses are variable and frequently incomplete. Increasing recognition of central sensitization and psychosocial contributors has led to the adoption of multidisciplinary treatment models. Clinical programs combining hormonal therapy, pain education, physical therapy, and behavioral interventions have demonstrated improvements in pain severity and functional outcomes, highlighting the limitations of pharmacologic therapy alone in managing chronic pelvic pain [22].

**Figure 2:**
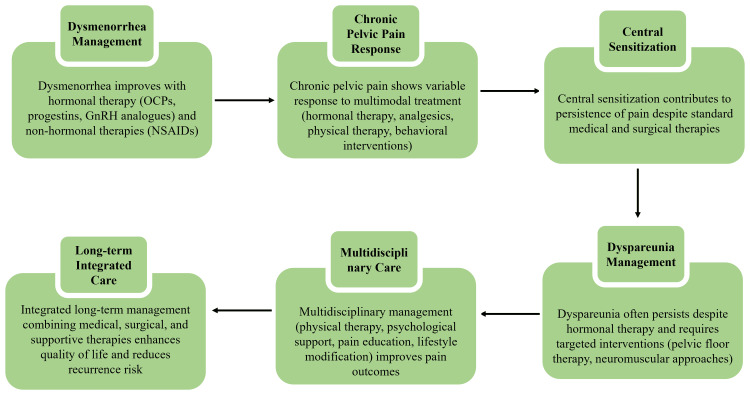
Multidimensional pain modulation in endometriosis under medical therapy. Created by authors using Microsoft PowerPoint.

Dyspareunia represents another complex and often persistent symptom, particularly in patients with deep infiltrating disease and pelvic floor dysfunction [20]. Hormonal suppression may reduce lesion-associated inflammation and pain; however, sexual pain frequently persists despite adequate control of other symptoms [21]. Evidence supports the use of adjunctive pelvic floor physical therapy to improve dyspareunia through neuromuscular re-education and reduction of pelvic floor hypertonicity [23]. These findings emphasize the importance of integrating medical and targeted non-hormonal therapies to address the multifaceted nature of endometriosis-related pain. The response pathways to pain following medical management of endometriosis are summarized in Figure 2.

Fertility outcomes associated with medical management

Treatment of endometriosis using medical therapy has complex implications for fertility, particularly due to the ovulation-suppressing effects of most hormonal treatments [8]. Although medical therapy is effective in controlling symptoms, it does not improve rates of spontaneous conception during treatment and may delay attempts at pregnancy [24]. Women may have a long history of prolonged hormonal suppression before fertility-oriented assessment, which may be distressing and could adversely affect reproductive timelines, particularly in those of advanced reproductive age [25]. Therefore, careful treatment planning is required in women desiring pregnancy, taking into account age, ovarian reserve, and disease severity [26].

Following discontinuation of medical therapy, fertility outcomes remain variable and are influenced by both disease-related and treatment-related factors. Sustained fertility benefit is not observed with prior hormonal suppression, and post-discontinuation conception rates vary, particularly in women with moderate to severe disease [27]. In the context of ART, endometriosis is associated with lower implantation and live birth rates compared with other infertility diagnoses, although outcomes vary depending on disease stage and treatment approach [28]. Moreover, women with endometriosis who achieve pregnancy, either spontaneously or through ART, have an increased risk of adverse obstetric outcomes, including preterm birth, highlighting the long-term reproductive impact of the disease beyond conception [29].

Clinical outcomes of surgical management

Surgical management plays a central role in the treatment of endometriosis, particularly in patients with severe symptoms, infertility, or disease refractory to medical therapy [13]. Laparoscopic interventions are the standard of care and are designed to remove identifiable disease, restore normal pelvic anatomy, and improve pain and fertility outcomes [15]. Although surgery is associated with significant symptom improvement, outcomes vary depending on the type of procedure, disease extent, and patient-specific factors.

Comparative evidence suggests that laparoscopic excision of endometriotic lesions is associated with superior clinical outcomes compared with ablation. Excision allows more complete removal of disease, including deep and fibrotic lesions, and is associated with longer-lasting pain relief and lower recurrence rates [24]. In contrast, ablation may be limited by incomplete treatment of deep lesions, contributing to persistent symptoms and earlier recurrence [23]. These differences are particularly relevant in deep endometriosis, where complete excision, although technically challenging, is associated with improved clinical outcomes [30].

Management of ovarian endometriomas presents additional challenges, particularly regarding preservation of ovarian reserve. Surgical cystectomy reduces pain and may improve spontaneous conception rates by restoring pelvic anatomy; however, it is associated with a decline in ovarian reserve, as evidenced by reduced anti-Müllerian hormone levels [27]. Therefore, surgical decision-making must balance potential fertility benefits against the risk of diminished ovarian function, especially in women planning ART [28].

Surgical management of deep infiltrating endometriosis is associated with improved reproductive outcomes in selected patients. Complete excision has been linked to higher rates of spontaneous conception, particularly in carefully selected individuals with significant infertility [20]. However, many patients still require postoperative ART to achieve pregnancy [30]. Despite surgical intervention, women with endometriosis remain at increased risk of adverse obstetric outcomes, including preterm birth, indicating persistent long-term reproductive risks. Table 2 summarizes the clinical implications of surgical management in endometriosis.

**Table 2:** Clinical outcomes of surgical management of endometriosis. AMH: anti-Müllerian hormone; DIE: deep infiltrating endometriosis; ART: assisted reproductive technology

Surgical aspect	Key findings	Clinical implications	References
Role of surgery	Surgical intervention improves pain and fertility outcomes, particularly in patients with severe symptoms, infertility, or disease refractory to medical therapy	Surgery remains a key treatment option when conservative management fails	[30]
Excision vs. ablation	Laparoscopic excision is associated with more durable pain relief and lower recurrence rates compared with ablation	Complete excision is preferred, especially for deep or fibrotic disease	[29]
Management of ovarian endometriomas	Cystectomy improves pain and may enhance spontaneous conception but is associated with a decline in ovarian reserve (decrease AMH levels)	Surgical decisions must balance pain and fertility benefits against ovarian reserve preservation	[27]
DIE	Complete excision of DIE is associated with improved spontaneous conception rates in selected patients	Multidisciplinary expertise is critical for optimal outcomes	[30]
Postoperative fertility outcomes	ART is often required following surgery when spontaneous conception does not occur	Integration of surgical and reproductive care improves fertility planning	[27]
Obstetric outcomes	Pregnancies in women with endometriosis have increased risk of adverse outcomes, including preterm birth, even after surgery	Long-term obstetric surveillance is warranted	[28]

Pain outcomes following surgical treatment

Surgical treatment of endometriosis is associated with significant short-term improvement in pain symptoms, including dysmenorrhea, chronic pelvic pain, and dyspareunia [23]. In the majority of patients, substantial symptom relief is achieved within the first postoperative year, reflecting effective removal of active disease and reduction of local nociceptive and inflammatory stimuli [27]. However, these benefits are not always sustained, and symptom recurrence is observed in a considerable proportion of patients over time [25]. Longitudinal studies indicate that recurrence of pain often parallels disease recurrence and is influenced by patient age, disease type, and postoperative management strategies [31]. Adjunctive postoperative hormonal suppression has been shown to reduce symptom recurrence and prolong pain-free intervals, supporting the use of combined treatment approaches [32].

The durability of pain relief following surgery is strongly influenced by the completeness of lesion excision [33]. Complete removal of endometriotic lesions, particularly in deep infiltrating endometriosis, is associated with improved long-term pain outcomes compared with partial resection or ablation [34]. Systematic reviews identify residual disease as a key predictor of persistent or recurrent pain, underscoring the importance of meticulous surgical technique and appropriate patient selection [35]. However, complete excision may require complex, multidisciplinary procedures and may be associated with increased perioperative risk, necessitating a balance between symptom control and surgical morbidity [34].

**Figure 3:**
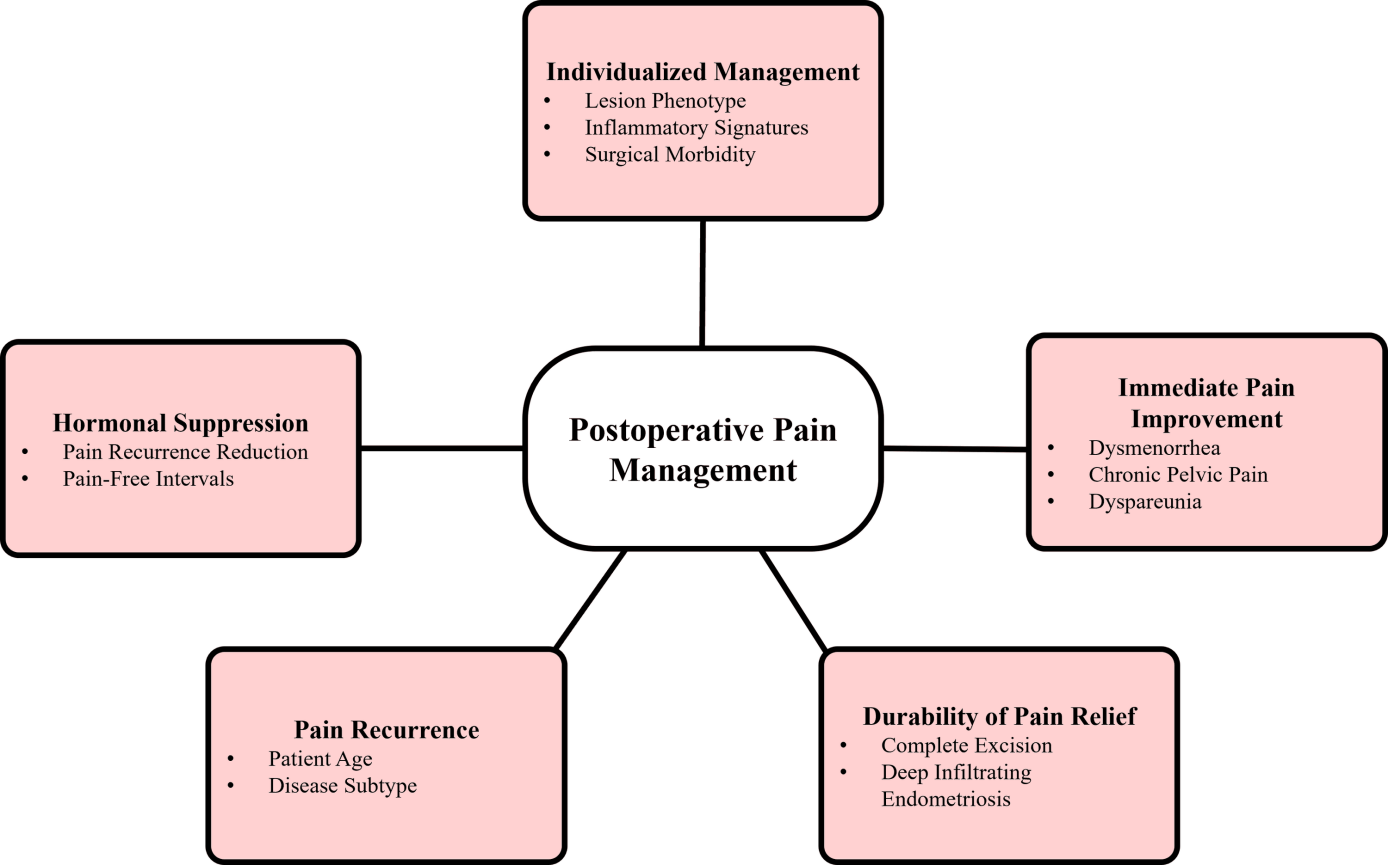
Endometriosis pain management framework. Created by authors using Microsoft PowerPoint.

Despite optimal surgical intervention, recurrence of pain remains common, reflecting the chronic and recurrent nature of endometriosis. Emerging evidence suggests that biological factors, including lesion phenotype, inflammatory profile, and molecular characteristics, may influence postoperative pain trajectories and recurrence risk [36]. These findings support the need for individualized postoperative management strategies that integrate surgical completeness, hormonal suppression, and long-term follow-up to optimize pain outcomes and improve QoL in women undergoing surgery for endometriosis [34]. Pathways in the management of postoperative pain in endometriosis are summarized in Figure 3.

Fertility outcomes following surgical management

Surgery is often considered a management option in infertile women with endometriosis, particularly in cases of anatomical distortion, presence of endometriomas, or deep infiltrating disease [33]. Restoration of normal pelvic anatomy and removal of inflammatory lesions are key mechanisms through which surgery may improve reproductive potential. Qualitative and clinical studies indicate that many women perceive surgery as a pivotal step in transitioning to fertility-focused care, especially after unsuccessful medical treatment [24].

Evidence suggests that surgical therapy, particularly complete excision of deep infiltrating endometriosis, is associated with improved spontaneous pregnancy rates in carefully selected patients [29]. Women undergoing extensive surgical removal of deep disease may achieve higher rates of spontaneous conception following surgery [30]. However, fertility outcomes after surgery are influenced by multiple factors, including patient age, disease severity, ovarian reserve, and extent of surgical intervention, highlighting the importance of individualized fertility counseling [26].

When spontaneous conception does not occur, ART plays an important role in post-surgical fertility management [25]. Meta-analyses demonstrate that endometriosis is associated with lower implantation, clinical pregnancy, and live birth rates compared with other causes of infertility, even after surgical treatment [27]. Moreover, pregnancies achieved either spontaneously or through ART in women with endometriosis are associated with an increased risk of adverse obstetric outcomes, including preterm birth, reflecting the persistent reproductive impact of the disease [29]. These findings indicate that while surgery may enhance fertility in selected patients, optimal outcomes are often achieved through integration of surgical intervention and assisted reproductive techniques within a multidisciplinary care model.

Recurrence rates after medical and surgical interventions

Endometriosis recurrence remains a significant challenge following both medical and surgical treatment, reflecting the chronic and relapsing nature of the disease [31]. The time to recurrence varies widely across studies and depends on treatment modality, disease subtype, and duration of follow-up [32]. Surgical intervention is generally associated with high rates of short-term symptom relief; however, recurrence rates increase over time, with long-term cohort studies reporting clinically significant recurrence of symptoms or disease several years after surgery [33]. Postoperative hormonal suppression is associated with delayed recurrence and a reduced likelihood of symptom relapse, supporting its role as an effective adjunct to surgical management [29].

Patterns of recurrence vary according to disease phenotype and completeness of surgical excision. Deep infiltrating endometriosis is associated with lower recurrence rates following complete excision compared with superficial disease, even in long-term follow-up studies [34]. In contrast, partial excision and conservative surgical approaches are associated with earlier and higher recurrence rates, emphasizing the importance of meticulous surgical technique and complete disease removal where feasible [35].

Several patient- and disease-related factors influence recurrence risk. Commonly reported predictors include younger age at surgery, advanced disease stage, presence of ovarian endometriomas, and absence of postoperative hormonal therapy [33]. Emerging evidence suggests that biological markers and PROMs may enhance risk stratification and facilitate earlier detection of recurrence, enabling more individualized postoperative management strategies [37]. The use of validated QoL and symptom assessment tools is increasingly recognized as essential for identifying clinically meaningful recurrence beyond imaging or surgical findings [38]. Table 3 summarizes recurrence patterns following medical and surgical management of endometriosis.

**Table 3:** Recurrence rates after medical and surgical management of endometriosis. PROMs: patient-reported outcome measures

Domain	Summary	Reference
Time to recurrence	Recurrence varies by treatment type, disease subtype, and follow-up duration; it increases over time after surgery	[32]
Postoperative hormonal therapy	Hormonal suppression prolongs recurrence-free intervals after surgery	[31]
Disease phenotype	Lower recurrence after complete excision of deep infiltrating disease	[34]
Surgical completeness	Incomplete excision is linked to earlier and higher recurrence	[32]
Key predictors	Younger age, severe disease, endometriomas, and no hormonal suppression increase risk	[33]
Emerging markers	Biomarkers and PROMs may improve recurrence prediction	[36]
Recurrence assessment	QoL and symptom tools essential beyond imaging alone	[38]

Quality of life and safety outcomes

QoL has become a key outcome in evaluating the effectiveness of endometriosis treatment, given the chronic nature of the disease and its substantial physical and psychosocial burden [33]. Validated endometriosis-specific and generic PROMs consistently demonstrate impairments in physical functioning, emotional well-being, sexual health, and social participation [39]. Medical and surgical therapies are associated with improvements in QoL when symptoms are effectively controlled; however, treatment tolerability and long-term adherence significantly influence sustained benefit [40]. These findings underscore the importance of integrating PROMs into clinical outcome assessment to capture meaningful patient-centered improvements beyond symptom reduction.

The impact of treatment on QoL is closely linked to safety and tolerability, particularly for medical therapies. Hormonal treatments, while effective in pain management, are associated with adverse effects such as irregular bleeding, weight changes, mood disturbances, vasomotor symptoms, and hypoestrogenic effects, especially with prolonged use of GnRH analogs [39]. Although many of these effects are reversible, cumulative side effects may lead to treatment discontinuation and reduced patient satisfaction [37]. Therefore, individualized counseling and careful selection of therapy are essential to balance efficacy with tolerability.

Surgical management also presents important safety considerations, particularly regarding postoperative complications and fertility outcomes. Although perioperative morbidity has decreased due to minimally invasive surgical techniques, risks of bleeding, infection, and organ injury persist, especially in complex procedures involving deep or ovarian disease [41]. A major concern is the impact of ovarian surgery on ovarian reserve, with studies demonstrating significant reductions in anti-Müllerian hormone levels following cystectomy for endometriomas [42]. The extent of ovarian reserve decline depends on factors such as lesion size, bilaterality, and surgical technique, emphasizing the need for fertility-sparing approaches where possible [43].

These considerations highlight the growing importance of fertility preservation in the management of endometriosis. Options such as oocyte or embryo cryopreservation should be discussed before surgical intervention, particularly in women with diminished ovarian reserve or future fertility intentions [44,45]. Overall, optimization of QoL in endometriosis requires a multidisciplinary approach that integrates symptom control, safety considerations, reproductive planning, and patient-reported outcomes into individualized care strategies.

Limitations and future directions

This comprehensive review has several limitations inherent to the current evidence base on endometriosis management. There is considerable heterogeneity across studies in terms of disease classification, patient populations, treatment regimens, and outcome assessment methods, which limits direct comparability of results. Non-uniform instruments are commonly used to measure pain, fertility, and QoL outcomes, and follow-up durations vary significantly, restricting interpretation of long-term effectiveness. In addition, a substantial proportion of evidence, particularly for surgical interventions, is derived from observational studies, making findings susceptible to selection bias and residual confounding. Diagnostic variability and delays further complicate outcome assessment, while underrepresentation of low- and middle-income settings may limit the generalizability of findings.

Future research should focus on well-designed prospective studies incorporating standardized disease phenotyping, validated outcome measures, and extended follow-up periods. Pain-refractory and fertility-focused populations require targeted comparative effectiveness trials evaluating medical, surgical, and combined treatment strategies. Advances in molecular profiling, imaging, and biomarker development may support more personalized treatment approaches and improve the prediction of treatment response and recurrence. Greater integration of patient-reported outcomes, multidisciplinary care models, and broader inclusion of diverse populations in research are essential to advance individualized, evidence-based management of endometriosis.

## Conclusions

Endometriosis is a chronic, heterogeneous condition requiring individualized, multidisciplinary management. Medical and surgical interventions are complementary in improving pain, fertility, and QoL. Medical therapy effectively controls symptoms but is limited by recurrence and lack of sustained fertility benefit. Surgical management offers significant benefits in selected patients, particularly with complete excision, but must be balanced against risks of recurrence, complications, and reduced ovarian reserve. Long-term management should incorporate patient-reported outcomes, fertility preservation, and multidisciplinary care to optimize outcomes.

## References

[1] Koninckx PR, Ussia A, Adamyan L, Tahlak M, Keckstein J, Wattiez A, Martin DC (2021). The epidemiology of endometriosis is poorly known as the pathophysiology and diagnosis are unclear. Best Pract Res Clin Obstet Gynaecol.

[2] Smolarz B, Szyłło K, Romanowicz H (2021). Endometriosis: epidemiology, classification, pathogenesis, treatment and genetics (review of literature). Int J Mol Sci.

[3] Koninckx PR, Ussia A, Keckstein J, Wattiez A, Adamyan L (2016). Epidemiology of subtle, typical, cystic, and deep endometriosis: a systematic review. Gynecol Surg.

[4] Horne AW, Missmer SA (2022). Pathophysiology, diagnosis, and management of endometriosis. BMJ.

[5] Cho JH, Feldman M (2015). Heterogeneity of autoimmune diseases: pathophysiologic insights from genetics and implications for new therapies. Nat Med.

[6] Bonavina G, Taylor HS (2022). Endometriosis-associated infertility: from pathophysiology to tailored treatment. Front Endocrinol (Lausanne).

[7] Pessoa de Farias Rodrigues M, Lima Vilarino F, de Souza Barbeiro Munhoz A, da Silva Paiva L, de Alcantara Sousa LV, Zaia V, Parente Barbosa C (2020). Clinical aspects and the quality of life among women with endometriosis and infertility: a cross-sectional study. BMC Womens Health.

[8] Szypłowska M, Tarkowski R, Kułak K (2023). The impact of endometriosis on depressive and anxiety symptoms and quality of life: a systematic review. Front Public Health.

[9] Warzecha D, Szymusik I, Wielgos M, Pietrzak B (2020). The impact of endometriosis on the quality of life and the incidence of depression-a cohort study. Int J Environ Res Public Health.

[10] Kalaitzopoulos DR, Samartzis N, Kolovos GN (2021). Treatment of endometriosis: a review with comparison of 8 guidelines. BMC Womens Health.

[11] Avery JC, Deslandes A, Freger SM (2024). Noninvasive diagnostic imaging for endometriosis part 1: a systematic review of recent developments in ultrasound, combination imaging, and artificial intelligence. Fertil Steril.

[12] Schreurs AM, Dancet EA, Apers S (2020). A systematic review and secondary analysis of two studies identifying demographic and medical characteristics determining patient-centeredness in endometriosis care as experienced by patients. Hum Reprod Open.

[13] Shafrir AL, Farland LV, Shah DK, Harris HR, Kvaskoff M, Zondervan K, Missmer SA (2018). Risk for and consequences of endometriosis: a critical epidemiologic review. Best Pract Res Clin Obstet Gynaecol.

[14] Brüggmann D, Elizabeth-Martinez A, Klingelhöfer D, Quarcoo D, Jaque JM, Groneberg DA (2016). Endometriosis and its global research architecture: an in-depth density-equalizing mapping analysis. BMC Womens Health.

[15] Falcone T, Flyckt R (2018). Clinical management of endometriosis. Obstet Gynecol.

[16] Allaire C, Bedaiwy MA, Yong PJ (2023). Diagnosis and management of endometriosis. CMAJ.

[17] Parasar P, Ozcan P, Terry KL (2017). Endometriosis: epidemiology, diagnosis and clinical management. Curr Obstet Gynecol Rep.

[18] Surrey ES (2023). GnRH agonists in the treatment of symptomatic endometriosis: a review. F S Rep.

[19] Kuan KK, Omoseni S, Tello JA (2023). Comparing ART outcomes in women with endometriosis after GnRH agonist versus GnRH antagonist ovarian stimulation: a systematic review. Ther Adv Endocrinol Metab.

[20] Kho KA, Shields JK (2020). Diagnosis and management of primary dysmenorrhea. JAMA.

[21] Carroquino-Garcia P, Jiménez-Rejano JJ, Medrano-Sanchez E, de la Casa-Almeida M, Diaz-Mohedo E, Suarez-Serrano C (2019). Therapeutic exercise in the treatment of primary dysmenorrhea: a systematic review and meta-analysis. Phys Ther.

[22] Westbay LC, Adams W, Kistner M, Brincat C, Bresler L, Yang LC, Fitzgerald CM (2021). Clinical outcomes of a multidisciplinary female chronic pelvic pain program. Female Pelvic Med Reconstr Surg.

[23] Schvartzman R, Schvartzman L, Ferreira CF, Vettorazzi J, Bertotto A, Wender MC (2019). Physical therapy intervention for women with dyspareunia: a randomized clinical trial. J Sex Marital Ther.

[24] Young K, Fisher J, Kirkman M (2016). Endometriosis and fertility: women's accounts of healthcare. Hum Reprod.

[25] Elizur SE, Mostafa J, Berkowitz E, Orvieto R (2025). Endometriosis and infertility: pathophysiology, treatment strategies, and reproductive outcomes. Arch Gynecol Obstet.

[26] Khan Z (2020). Fertility-related considerations in endometriosis. Abdom Radiol (NY).

[27] Hamdan M, Omar SZ, Dunselman G, Cheong Y (2015). Influence of endometriosis on assisted reproductive technology outcomes: a systematic review and meta-analysis. Obstet Gynecol.

[28] González-Comadran M, Schwarze JE, Zegers-Hochschild F, Souza MD, Carreras R, Checa MÁ (2017). The impact of endometriosis on the outcome of assisted reproductive technology. Reprod Biol Endocrinol.

[29] Pérez-López FR, Villagrasa-Boli P, Muñoz-Olarte M, Morera-Grau Á, Cruz-Andrés P, Hernandez AV (2018). Association between endometriosis and preterm birth in women with spontaneous conception or using assisted reproductive technology: a systematic review and meta-analysis of cohort studies. Reprod Sci.

[30] Grigoriadis G, Daniilidis A, Merlot B (2024). Surgical treatment of deep endometriosis: impact on spontaneous conception. Best Pract Res Clin Obstet Gynaecol.

[31] Zakhari A, Delpero E, McKeown S, Tomlinson G, Bougie O, Murji A (2021). Endometriosis recurrence following post-operative hormonal suppression: a systematic review and meta-analysis. Hum Reprod Update.

[32] Nirgianakis K, Ma L, McKinnon B, Mueller MD (2020). Recurrence patterns after surgery in patients with different endometriosis subtypes: a long-term hospital-based cohort study. J Clin Med.

[33] Bozdag G (2015). Recurrence of endometriosis: risk factors, mechanisms and biomarkers. Womens Health (Lond).

[34] Ianieri MM, Mautone D, Ceccaroni M (2018). Recurrence in deep infiltrating endometriosis: a systematic review of the literature. J Minim Invasive Gynecol.

[35] Ceccaroni M, Bounous VE, Clarizia R, Mautone D, Mabrouk M (2019). Recurrent endometriosis: a battle against an unknown enemy. Eur J Contracept Reprod Health Care.

[36] Holdsworth-Carson SJ, Chung J, Machalek DA (2024). Predicting disease recurrence in patients with endometriosis: an observational study. BMC Med.

[37] Nicolas-Boluda A, Oppenheimer A, Bouaziz J, Fauconnier A (2021). Patient-reported outcome measures in endometriosis. J Clin Med.

[38] Bourdel N, Chauvet P, Billone V, Douridas G, Fauconnier A, Gerbaud L, Canis M (2019). Systematic review of quality of life measures in patients with endometriosis. PLoS One.

[39] Berlanda N, Somigliana E, Viganò P, Vercellini P (2016). Safety of medical treatments for endometriosis. Expert Opin Drug Saf.

[40] Barbara G, Buggio L, Facchin F, Vercellini P (2021). Medical treatment for endometriosis: tolerability, quality of life and adherence. Front Glob Womens Health.

[41] Goodman LR, Goldberg JM, Flyckt RL, Gupta M, Harwalker J, Falcone T (2016). Effect of surgery on ovarian reserve in women with endometriomas, endometriosis and controls. Am J Obstet Gynecol.

[42] Chiang HJ, Lin PY, Huang FJ, Kung FT, Lin YJ, Sung PH, Lan KC (2015). The impact of previous ovarian surgery on ovarian reserve in patients with endometriosis. BMC Womens Health.

[43] Roman H (2018). Endometriosis surgery and preservation of fertility, what surgeons should know. J Visc Surg.

[44] Ashrafi M, Arabipoor A, Hemat M, Salman-Yazdi R (2019). The impact of the localisation of endometriosis lesions on ovarian reserve and assisted reproduction techniques outcomes. J Obstet Gynaecol.

[45] Sönmezer M, Taşkın S (2015). Fertility preservation in women with ovarian endometriosis. Womens Health (Lond).

